# Clinically defined aspiration pneumonia is an independent risk factor associated with long-term hospital stay: a prospective cohort study

**DOI:** 10.1186/s12890-023-02641-y

**Published:** 2023-09-18

**Authors:** Takao Wakabayashi, Sugihiro Hamaguchi, Konosuke Morimoto, on behalf of the Adult Pneumonia Study Group – Japan, Motoi Suzuki, Eiichiro Sando, Bhim Gopal Dhoubhadel, Akitsugu Furumoto, Masayuki Ishida, Tomoko Ishifuji, Satoshi Kakiuchi, Shungo Katoh, Emi Kitashoji, Takaharu Shimazaki, Masahiro Takaki, Kiwao Watanabe, Lay Myint Yoshida, Koya Ariyoshi, Kota Sasaki, Hidenori Katsura, Hiroki Nanba, Masahiko Abe, Makito Yaegashi, Masahiro Aoshima, Naoto Hosokawa, Norihiro Kaneko, Hidenori Katsura, Naoko Katsurada, Kei Nakashima, Yoshihito Otsuka, Daisuke Suzuki, Kenzo Tanaka, Naohisa Hamashige, Masayuki Chikamori, Hiroshi Nakaoka, Norichika Asoh, Hiroyuki Ito, Kei Matsuki, Yoshiko Tsuchihashi, Shimpei Morimoto, Shuntaro Sato

**Affiliations:** 1 Department of General and Emergency Medicine, Japan Community Healthcare Organization Sapporo Hokushin Hospital, 2-1,2-Jo,6-Chome, Atsubetsu-Cho, Atsubetsu-Ku, Sapporo, 004-8618 Japan; 2 Department of General Internal Medicine, Ebetsu City Hospital, Hokkaido, Japan; 3 Department of General Internal Medicine, Fukushima Medical University, 1, Hikarigaoka, Fukushima, 960-1295 Japan; 4 Department of Respiratory Infections, Institute of Tropical Medicine, Nagasaki University, 1-12-4 Sakamoto, Nagasaki, 852-8523 Japan

**Keywords:** Aspiration pneumonia, Community-acquired pneumonia, Healthcare-associated pneumonia, Long-term hospital stay, Rehabilitation

## Abstract

**Background:**

Long-term hospital stay is associated with functional decline in patients with pneumonia, especially in the elderly. Among elderly patients with pneumonia, aspiration pneumonia is a major category. Clinical definition is usually used because it can occur without apparent aspiration episodes. It is still not clear whether a long-term hospital stay is due to aspiration pneumonia itself caused by underlying oropharyngeal dysfunction or simply due to functional decline in elderly patients with multiple comorbidities during acute infection. The aim of this study is to identify whether clinically defined aspiration pneumonia itself was associated with a long-term hospital stay.

**Methods:**

A prospective observational study on community-acquired (CAP) or healthcare-associated pneumonia (HCAP) was conducted from January 2012 through January 2014. Aspiration pneumonia was clinically defined as pneumonia not only occurring in patients after documented aspiration episodes, but also occurring in those with underlying oropharyngeal dysfunction: chronic disturbances of consciousness and/or chronic neuromuscular diseases. We defined thirty-day hospital stay as a long-term hospital stay and compared it with logistic regression analysis. Potential confounders included age, sex, HCAP, body mass index (BMI), long-term bed-ridden state, heart failure, cerebrovascular disorders, dementia, antipsychotics use, hypnotics use, and CURB score which is a clinical prediction tool used to assess the severity, standing for; C (presence of Confusion), U (high blood Urea nitrogen level), R (high Respiratory rate), and B (low Blood pressure). In a sub-analysis, we also explored factors associated with long-term hospital stay in patients with aspiration pneumonia.

**Results:**

Of 2,795 patients, 878 (31.4%) had aspiration pneumonia. After adjusting potential confounders, the aspiration pneumonia itself was significantly associated with long-term hospital stay (adjusted odds ratio 1.44; 95% confidence interval 1.09—1.89, *p* < 0.01), as were higher age, male sex, high CURB score, HCAP, low BMI, heart failure, cerebrovascular disease, and antipsychotics use. Sub-analysis revealed factors associated with long-term hospital stay in the aspiration pneumonia, which included male sex, and multi-lobar chest X-ray involvement.

**Conclusions:**

Clinically defined aspiration pneumonia itself was independently associated with long-term hospital stay. This result could potentially lead to specific rehabilitation strategies for pneumonia patients with underlying oropharyngeal dysfunction.

**Supplementary Information:**

The online version contains supplementary material available at 10.1186/s12890-023-02641-y.

## Background

Pneumonia is one of the most common clinical syndromes and an important public health issue with serious personal and economic ramifications. Pneumonia is currently the third leading cause of mortality in Japan. There are several clinical classifications of pneumonia based on setting, clinical characteristics, causative microbes, and prognosis [1]. Aspiration pneumonia is a distinct and clinically useful category of this disease. This condition is defined as an acute lung infection that develops in response to significant aspiration of oropharyngeal or upper gastrointestinal contents [2]. However, it is sometimes difficult to prove the evidence of aspiration in patients with pneumonia. One previous study used the definition of aspiration pneumonia as pneumonia associated with either witnessed aspiration or risk factors for aspiration and showed that as many as 23% of community-acquired pneumonia (CAP) were aspiration pneumonia [3]. Likewise, another study used the definition of pneumonia in patients with a predisposition to aspiration and indicated that up to 60% of hospitalized patients with CAP were found to have aspiration pneumonia [4]. Therefore, aspiration pneumonia is often clinically defined as pneumonia occurring in patients with risk factors for aspiration as well as that occurring after a witnessed aspiration episode [5]. As a result, clinically defined aspiration pneumonia is a familiar condition to most clinicians.

In recent years, hospitalization costs associated with aspiration pneumonia have been increasing in the United States [6]. Previous studies have revealed that a single episode of acute illness involving hospitalization can be accompanied by functional decline, deterioration in performance of activities of daily living (ADL), and the need for long-term care [7–12]. A decline in capacity to perform ADL was particularly notable for elderly patients who were hospitalized due to lower respiratory infections [13]. Long-term hospital stay has been associated with functional decline [14] and can lead to a substantial decrease in quality of life (QOL) [15, 16]. In this way, long-term hospital stay with aspiration pneumonia may also cause a large amount of medical expenses and lower the ADL and QOL of hospitalized patients.

It is still not clear whether a long-term hospital stay is simply due to functional decline in elderly patients with acute infection or rather due to pneumonia in patients with underlying oropharyngeal dysfunctions, which are the risk factors for aspiration. The answer to this question can provide a better focus on specific rehabilitation strategies, such as swallowing training. Therefore, the goal of our study is to clarify whether or not aspiration pneumonia per se, the equivalent of pneumonia with underlying oropharyngeal dysfunction, represents an independent risk factor associated with long-term hospital stay. In this study, we defined aspiration pneumonia clinically and we conducted a prospective cohort study to identify the relationship between aspiration pneumonia and long-term hospital stay among patients, including a substantial number of elderly persons, with CAP or healthcare-associated pneumonia (HCAP). As a sub-analysis, we explored the potential risk factors that might be independently associated with long-term hospital stay in the group of patients with aspiration pneumonia.

## Method

### Design and setting / study population

This research was performed as a part of a multicenter prospective study of adult pneumonia by Adult Pneumonia Study Group-Japan (APSG-J) [17]. The APSG-J study enrolled patients from four community-based hospitals in Japan from September 2011 to January 2014. We collected information from the medical records of patients diagnosed with CAP and HCAP including age, sex, diagnosis of aspiration pneumonia, laboratory data, comorbidities, and measures of disease severity. Hospital physicians provided a clinical diagnosis that included the presence or absence of aspiration as indicated on the patient data form.

All hospitalized patients and outpatients aged 15 years or older were screened by hospital physicians; patients who met the standard case definition were identified. Patients who fulfilled all the following criteria were enrolled in the study: (1) age ≥ 15 years, (2) symptoms indicative of pneumonia (e.g., fever, cough, sputum, pleuritic chest pain, and dyspnea), and (3) evidence of new-onset pulmonary infiltrates on chest radiography and/or computed tomography scan films that were consistent with this diagnosis. All the enrolled cases were classified as CAP or HCAP according to the definitions in the American Thoracic Society (ATS) / Infectious Diseases Society of America guidelines [18, 19]. Patients diagnosed with HCAP were those who were hospitalized for ≥ 2 days in the preceding 90 days, who were residents of a nursing home or extended-care facility, those receiving home infusion therapy (including antibiotics) or who underwent chronic dialysis within 30 days of diagnosis, and those treated with home wound care or who have a family member diagnosed with multi-resistant pathogens. Patients who developed signs and symptoms within 48 h after admission were diagnosed with hospital-acquired pneumonia; these patients were excluded from this research study. Likewise, repeated episodes of pneumonia in a single patient within a 2-week period were regarded as a single episode.

### Exposure

As mentioned earlier, defining aspiration pneumonia is challenging because it is often difficult to prove the evidence of aspiration in patients with pneumonia and a significant proportion of patients have pneumonia due to so-called silent aspiration [20, 21]. Therefore, many studies used clinical definition: pneumonia occurring in patients with risk factors for aspiration as well as with aspiration episodes [22]. Accordingly, we defined aspiration pneumonia as pneumonia not only occurring in patients after documented episodes of aspiration, but also occurring in those with underlying oropharyngeal dysfunction: chronic disturbances of consciousness and/or chronic neuromuscular diseases [23–25]. As confounding factors for long-term hospital stay, we selected age, sex, a diagnosis of HCAP, CURB score, body mass index (BMI), long-term bed-ridden state, heart failure, cerebrovascular disorders, dementia, use of antipsychotic and use of hypnotic drugs. The confounding factors were selected as variables associated with both exposure and outcome based on previous studies and medical findings. Age is included in CURB65 [26], a severity classification of pneumonia, and is known to be associated with risk of mortality [27]. Male sex was shown to be associated with high mortality among pneumonia patients [28]. We used the CURB score [29] excluding the age factor from CURB65 in order to adjust, more accurately the effect of age. CURB score is a clinical prediction tool used to assess the severity of CAP and guide the decision-making tool regarding the need for a hospital stay. CURB stands for; C (presence of Confusion), U (blood Urea nitrogen level ≥ 7 mmol/L), R (Respiratory rate > 30 per minute), and B (Blood pressure < 90 mmHg in systole or ≤ 60 mmHg in diastole). The score is calculated as the summation of four findings with one point given for each finding present (range 0–4 points). The total score of these helps in determining the risk and severity of pneumonia. Higher scores indicate a greater likelihood of severe pneumonia [29]. The score of CURB 2 or higher is defined as severe pneumonia [29]. The HCAP [19, 30], BMI [31] and ADL [32] have been associated with pneumonia-associated mortality; as such, we included these factors as potential confounders as a way to adjust for patient disease severity. The HCAP classification has recently been deleted from the guidelines. However, we consider this classification as a simple indicator for estimating disease severity. A long-term bed ridden was adopted as a surrogate index for ADL. In comorbidity and drug, heart failure was shown to be a risk of mortality in CAP patients [33] and cerebrovascular disorder [34], dementia [35], use of antipsychotic drugs [36], and use of hypnotic drugs [37] have all been associated with aspiration pneumonia in previous studies.

For sub-analysis, we sought to identify independent variables associated with long-term hospital stay due to aspiration pneumonia. We selected potential risk factors for long-term hospital stay that showed a significant difference in the univariate analysis between the aspiration pneumonia group and the other pneumonia group. For this analysis, the aforementioned factors were evaluated as potential independent variables, and long-term hospital stay was introduced as the dependent variable.

### Outcomes

The primary outcome was a long-term hospital stay. We defined the long-term hospital stay as thirty-day or longer hospitalization. The term, thirty-day, was often used as the standard criterion regarding mortality and readmission in various studies.

In the sub-analysis, we aimed to explore the potential independent factors associated with long-term hospital stay in patients with aspiration pneumonia.

### Statistical analysis

Baseline characteristics were presented using standard descriptive statistics, including means for age and laboratory data, median with interquartile range (IQR) for other continuous variables and percentages for categorical variables. For the primary outcome, adjusted odds ratio (AOR) and 95% confidence interval (CI) for the likelihood of having long-term hospital stay were estimated using a multiple logistic regression model. In the logistic regression model, we adjusted for the clinically relevant potential confounding factors. As we could not feasibly neglect missing values for certain variables, we encoded these missing values as "unknown states" and included them in the analysis. This facilitated inclusion of all patients in the primary analysis. For the sub-analysis, we compared continuous variables between groups with a two-sample t-test or non-parametric Mann–Whitney test and Fisher’s exact test was used to compare the participants’ clinical characteristics for categorical data. We performed logistic analysis in order to identify significant risk factors associated with long-term hospital stay in the aspiration pneumonia patient cohort. A *p*-value of < 0.05 was considered to be statistically significant. All analyses were performed with SPSS version 22.0 for Windows (SPSS Inc, Chicago, IL, USA).

### Ethical consideration

The study was conducted in accordance with the Guideline for Ethical Aspects in Epidemiological Study (Ministry of Health, Labour and Welfare, Japan 2008). This study was approved by the Institutional Review Board of Tropical Medicine at Nagasaki University and the institutional review board of each participating hospital; Ebetsu City Hospital, Kameda Medical Centre, Chikamori Hospital, and Juzenkai Hospital. We used a standardized questionnaire form to provide patients and their guardians with necessary information. Written informed consent to participate in this study was obtained from participants or their guardians. Investigators maintain all patient-related datasets in password-protected systems and present the data in this manuscript while maintaining the anonymity of the study participants.

## Results

### Baseline characteristics

**Fig. 1:**
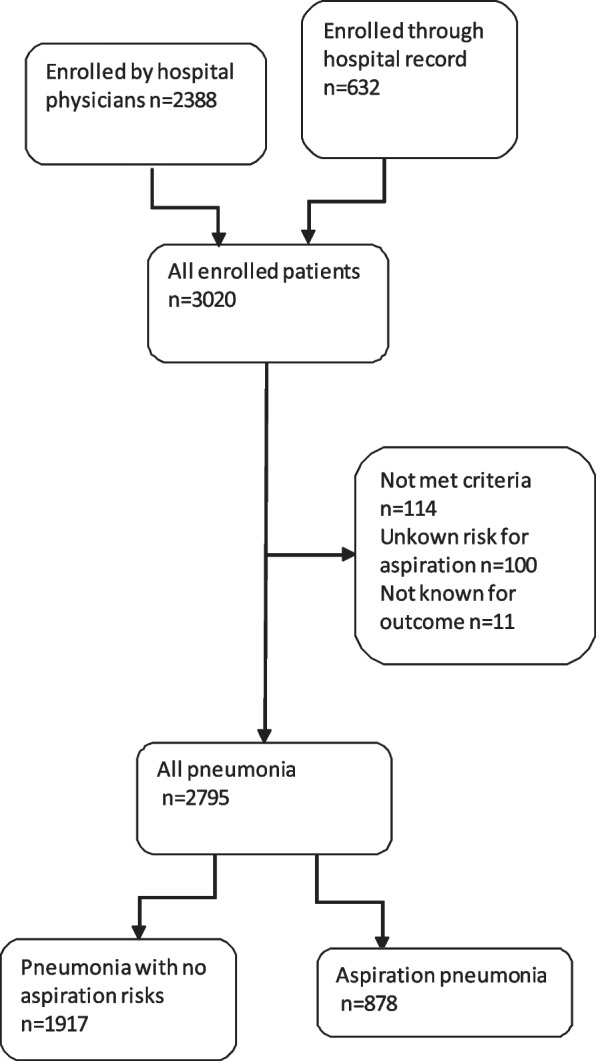
Selection of participants

The original cohort study included 3,020 patients. Of these, 225 patients were excluded; 114 patients did not meet the inclusion criteria, 100 patients lacked the information on aspiration risk factors, and 11 patients lacked outcome data. As such, we enrolled 2,795 patients with diagnoses of CAP or HCAP in this study (Fig. 1). Of this group, 878 patients were included in the aspiration pneumonia group (31.4%); 703 of these patients had experienced episodes of aspiration, 151 had chronic disturbances of consciousness, and 179 were diagnosed with chronic neuromuscular diseases that did not include previous stroke or dementia. Some patient cases included more than one risk factor.　Baseline characteristics are summarized in Table 1. The mean age was 73.4 years; 59.5% of the participants were males.95.7% of aspiration pneumonia patients were hospitalized, and the median length of hospital stay was 16 days (IQR; 10-27 days).

**Table 1:** Baseline characteristics

	Aspiration pneumonia Group *n* = 878 (%)	No aspiration risks Group *n* = 1,917 (%)	All Participants *n* = 2,795 (%)
**Basic information**			
Age(y),mean	78.9	70.9	73.4
Age(75 or more) (%)	653 (74.4)	1020(53.2)	1,673(59.9)
Sex (male) (%)	518( 59.0)	1,145(59.7)	1,663(59.5)
Number of hospitalized patient (%)	840(95.7)	1,300(67.8)	2140(76.6)
Length of hospital stay (days),IQR	19 (12–31) (*n* = 840)	14 (9–24) (*n* = 1,300)	16 (10–27) (*n* = 2,140)
HCAP (%)	539(61.4)	428(22.3)	967(34.6)
CURB score (2 < or =)	283 (37.7) (*n* = 751)	289 (20.0) (*n* = 1,448)	751 (34.2) (*n* = 2,199)
Long-term bed ridden (%)	225(25.7)	86(4.4)	311(11.1)
BMI(< 18.5) (%)	157/393(39.9)	521/1,893(27.5)	678/2,286(29.7)
**Vital sign**			
Acute consiousness disorder (%)	300/876(34.2)	169/1,911(8.8)	469/2,787(16.8)
**Chest X-ray**			
Multilobar chest X-ray involvement (%)	298(33.9)	445(23.2)	743(26.6)
**Laboratory data**			
BUN (mg/dl),mean (n)	24.2 (*n* = 875)	20.3 (*n* = 1855)	21.6 (*n* = 2730)
Albumin,mean (n)	3.3 (*n* = 837)	3.5 (*n* = 1731)	3.4 (*n* = 2568)
**Comorbidities**			
Heart failure (%)	165(18.8)	246(12.8)	411(14.7)
Ischemic heart disease (%)	106(12.1)	210(11.0)	316(11.3)
Liver disease (%)	49(5.6)	89(4.6)	138(4.9)
Kidney disease (%)	98(11.2)	203(10.6)	301(10.8)
Dementia (%)	277(31.5)	153(8.0)	430(15.4)
Cerebrovascular disease (%)	351(40.0)	294(15.3)	645(23.1)
Malignancy (%)	162(18.5)	325(16.7)	487(17.4)
Chronic respiratory disease (%)	149(17.0)	452(23.6)	601(21.5)
**Drugs**			
Use of antipsychotics (%)	146(16.6)	153(8.0)	299(10.7)
Use of hypnotics (%)	143(16.3)	172(9.0)	315(11.3)
Use of antiacids (%)	307(35.0)	512(26.7)	819(29.3)
Use of ACE inhibitor (%)	41(4.7)	106(5.5)	147(5.3)

Values for categorical variables indicate percentage; values for continuous variables indicate medican

*IQR* interquartile range, *HCAP* health-care associated pneumonia, *CURB* Confusion, Blood urea nitrogen > 20 mg/dl,Respiratory rate > 30 per minute, Blood pressure < 90 mmHg in systole or <  = 60 mmHg in diastole

*BMI* Body mass index is the weight in kilograms divided by the square of the height in meters

*ACE* angiotensin-converting-enzyme, *BUN* Blood urea nitrogen

### Primary outcome

We estimated AOR of long-term hospital stay associated with aspiration pneumonia (Table 2). After adjusting the potential confounders, the aspiration pneumonia is independently a risk factor of long-term hospital stay (AOR 1.44; 95% CI 1.09—1.89, *p* < 0.01). Higher age (AOR 1.01; 95% CI 1.003—1.02, *p* < 0.05), male sex (AOR 1.40; 95% CI 1.06—1.82, *p* < 0.05), CURB score (2 < or =) (AOR 1.52; 95% CI 1.17—1.99, *p* < 0.01), diagnosis of HCAP (AOR 1.48; 95% CI 1.12—1.96, *p* < 0.01), low BMI (AOR 0.95; 95%CI 0.91—0.98, *p* < 0.01), heart failure (AOR 1.44; 95% CI 1.05—1.96, *p* < 0.01), cerebrovascular disease (AOR 1.35; 95% CI 1.02—1.78, *p* < 0.01), and use of antipsychotics (AOR 1.50; 95% CI 1.05—2.15, *p* < 0.05) were suggested to be the risk of long-term hospital stay.

**Table 2:** Adjusted ORs of long-term hospital stay

	Adjusted		
	OR	95%CI	*p*-value
Aspiration pneumonia	1.44	1.09–1.89	< 0.01
Age per y	1.01	1.003–1.02	< 0.05
Sex (male)	1.40	1.06–1.82	< 0.05
CURB score (2 < or =)	1.52	1.17–1.99	< 0.01
HCAP	1.48	1.12–1.96	< 0.01
BMI (score per 1)	0.95	0.91–0.98	< 0.01
Long-term bed ridden	0.98	0.68–1.41	0.90
Heart failure	1.44	1.05–1.96	< 0.05
Cerebrovascular disease	1.35	1.02–1.78	< 0.05
Dementia	1.23	0.89–1.71	0.21
Use of antipsychotics	1.50	1.05–2.15	< 0.05
Use of hypnotics	1.05	0.73–1.51	0.81

*OR* odds ratio, *CI* confidence interval

*CURB* Confusion, Blood urea nitrogen > 20 mg/dl,Respiratory rate > 30 per minute, Blood pressure < 90 mmHg in systole or <  = 60 mmHg in diastole

*HCAP* health-care associated pneumonia

*BMI* Body mass index is the weight in kilograms divided by the square of the height in meters

### Sub analysis

Of the 878 patients diagnosed with aspiration pneumonia, 229 (26.1%) were among those who experienced a long-term hospital stay. In the univariate analysis, there was a significant difference in male sex, CURB score (2 < or =), multi-lobar chest X-ray involvement, albumin (per 1 g/dl), liver disease, kidney disease and use of antipsychotics between two groups (Supplemental Table 1). We added variables related to age to these factors and we set them as confounding factors. 

In this sub-analysis, we found that male sex (AOR 1.52; 95%CI 1.06—2.18, *p* < 0.05), and multi-lobar chest X ray involvement (AOR 1.48; 95% CI 1.04—2.12, *p* < 0.05) were among the independent risk factors associated with a longer hospital stay among patients diagnosed with aspiration pneumonia (Table 3).

**Table 3:** Adjusted ORs of Risk factors for 30-days hospital stay in the aspiration pneumonia

	Adjusted		
	OR	95%CI	*p*-value
Age (per 1)	1.01	0.99–1.03	0.07
Sex (male)	1.52	1.06–2.18	< 0.05
CURB score (2 < or =)	1.39	0.98–1.98	0.07
Multi-lobar chest X-ray involvement	1.48	1.04–2.12	< 0.05
Albumin (per 1 g/dl)	0.81	0.59–1.11	0.20
Liver disease	1.33	0.65–2.72	0.44
Kidney disease	1.53	0.92–2.54	0.10
Use of antipsychotics	1.55	0.99–2.44	0.06

*OR* odds ratio, *CI* confidence interval

*CURB* Confusion, Blood urea nitrogen > 20 mg/dl,Respiratory rate > 30 per minute, Blood pressure < 90 mmHg in systole or <  = 60 mmHg in diastole

## Discussion

We identified clinically defined aspiration pneumonia itself as an independent risk factor associated with long-term hospital stay. We also identified that male sex, and multi-lobar chest X ray involvement were associated with long-term hospital stay among patients with aspiration pneumonia in an exploratory sub-analysis.

The reason why aspiration pneumonia itself is associated with long-term hospital stay may be that dysphagia in pneumonia patients is an inhibitory factor of discharge from hospital. Previous studies also revealed that aspiration pneumonia was associated with an increase in recurrent pneumonia [38]. This condition may result from repeated aspiration, either silent or in evidence, which may result in long-term hospital stay even when the pneumonia itself is adequately treated. Therefore, both pre-, in-, and post-hospital interventions are important.

The most important pre-hospital intervention may be disease prevention. Implementation of preventive measures, including drug therapy, vaccination and/or rehabilitation, is a critical intervention, notably in patients with risk factors for aspiration. As an in-hospital intervention, in addition to adequate antibiotic therapy, early rehabilitation and physical therapy have been shown to shorten the length of hospital stay in patients with dysphagia [39]. Follow-up management strategies such as newer rehabilitation approaches of stimulation techniques for patients with aspiration pneumonia due to dysphagia have already been recommended [40]. In recent studies, the thickness of the erector spine muscles has been showed to be associated with prognosis of many diseases, including aspiration pneumonia [41–43]. Strengthening the erector spine muscles may also be a specific rehabilitation strategy to prevent aspiration pneumonia or shorten the length of hospital stay.

The strong point of our research is that we defined aspiration pneumonia clinically not only as pneumonia caused by apparent aspiration episodes but also as pneumonia with aspiration risks. By so doing, we could include aspiration pneumonia without detectable aspiration episodes, known as “silent aspiration” in our study patients. In real practice, aspiration pneumonia often occurs due to silent aspiration. Excluding those cohort patients could lead to different results and interpretations. In fact, when we specifically analyzed only pneumonia after apparent aspiration episodes (*n* = 703), no significant difference was observed regarding the primary outcome (AOR 1.18; 95%CI 0.86—1.62, *p* = 0.30).

In addition to aspiration pneumonia itself, our study also suggests that higher age, male sex, high CURB score, diagnosis of HCAP, low BMI, heart failure, cerebrovascular disease and use of antipsychotics are also independently associated with long-term hospital stay. Higher age, male sex, high CURB score, diagnosis of HCAP, and low BMI may indicate the severity of the disease. The presence of heart failure and cerebrovascular disease may prolong the care of pneumonia itself or prolong the time required for rehabilitation. Use of antipsychotics may cause impaired consciousness and delirium, which may extend the length of hospital stay.

In the sub-analysis, we identified that male sex, and multi-lobar chest X ray involvement were the risks of long-term hospital stay in the aspiration pneumonia group. As mentioned above, male sex was shown to be associated with mortality. In some studies, multi-lobar chest X-ray involvement [44, 45] were used as a tool for prediction of the need for hospitalization among patients diagnose with pneumonia. The sub-analysis outcomes might be used to predict the risk of a long-term hospital stay among those patients diagnosed with aspiration pneumonia.

Our study has some limitations. The first limitation relates to the functional and morphological definition of aspiration pneumonia. In this study, we did not confirm the dysphagia using a laryngeal endoscopic procedure. Thus, the definition of clinically defined aspiration pneumonia does not include the confirmation of functional abnormalities. However, in real clinical practice, we do not always use laryngeal endoscopy to diagnose aspiration pneumonia. Therefore, we used the clinical definition, pneumonia occurring in patients with risk factors for aspiration. The latest review also defines aspiration pneumonia as the confirmation of inflammatory findings in the lungs plus evidence of overt aspiration or existence of a condition in which aspiration is strongly suspected [21]. The review pointed out that since apparent aspiration is difficult to be confirmed directly, quality journals have accepted this clinical definition.

Next, we selected potential confounding factors when generating adjustments in the multivariate analysis. However, there is a possibility that unmeasured confounding factors influence the outcomes. In particular, social factors, such as annual income and public support, can affect the length of hospital stay. However, thanks to the universal healthcare insurance system of Japan, financial factors for medical care may minimally affect the length of hospital stay in Japan.

Finally, we note that the relationship between aspiration pneumonia and the primary outcome was represented by the AOR 1.44 with 95% CI of 1.09–1.89. The magnitude of aspiration pneumonia itself as a factor for a long-term hospital stay was not very outstanding. Comprehensive management including those for other modifiable factors is necessary to prevent long-term hospital stay in patients with aspiration pneumonia.

## Conclusions

In this observational cohort study, we found that clinically defined aspiration pneumonia itself was an independent risk factor associated with long-term hospital stay. We also identified that male sex, and multi-lobar chest X-ray involvement were associated with long-term hospital stay among patients with aspiration pneumonia.

### Supplementary Information

**Additional file 1:** **Supplemental Table 1.** Comparison between long-term hospital stay group and the other group among aspiration pneumonia patients.

## Data Availability

The original data in the current study can be available from the corresponding author on reasonable request.

## Abbreviations

ADL: Activities of daily living
AOR: Adjusted odds ratio
ATS: American Thoracic Society
BMI: Body mass index
CAP: Community-acquired pneumonia
CI: Confidence interval
CURB: Confusion, elevated blood urea nitrogen, tachypnea, hypotension
CURB65: Confusion, elevated blood urea nitrogen, tachypnea, hypotension, and age ≥ 65
HCAP: Healthcare-associated pneumonia
IQR: Interquartile range
QOL: Quality of life
